# One-Step Hydrothermal Strategy for Preparation of a Self-Cleaning TiO_2_/SiO_2_ Fiber Membrane toward Oil-Water Separation in a Complex Environment

**DOI:** 10.3390/membranes13050514

**Published:** 2023-05-15

**Authors:** Yinghao Lin, Atian Xie, Jian Xu, Changguo Xue, Jiuyun Cui, Jianming Pan

**Affiliations:** 1 School of Materials Science and Engineering, Anhui University of Science and Technology, Huainan 232001, China; 2 School of Chemistry and Chemical Engineering, Jiangsu University, Zhenjiang 212013, China; 3 Jiangsu Agrochem Laboratory Co., Ltd., Changzhou 213022, China

**Keywords:** superhydrophilicity/underwater superoleophobicity, fiber membrane, TiO_2_, oil-water separation, self-cleaning

## Abstract

Oil pollution caused by a large number of industrial activities and oil spill accidents has posed serious harm to the environment and human health. However, some challenges remain with the existing separation materials, such as poor stability and fouling resistance. Herein, a TiO_2_/SiO_2_ fiber membrane (TSFM) was prepared by a one-step hydrothermal method for oil-water separation in acid, alkali, and salt environments. The TiO_2_ nanoparticles were successfully grown on the fiber surface, endowing the membrane with superhydrophilicity/underwater superoleophobicity. The as-prepared TSFM exhibits high separation efficiency (above 98%) and separation fluxes (3016.38–3263.45 L·m^−2^·h^−1^) for various oil-water mixtures. Importantly, the membrane shows good corrosion resistance in acid, alkaline, and salt solutions and still maintains underwater superoleophobicity and high separation performance. The TSFM displays good performance after repeated separation, demonstrating its excellent antifouling ability. Importantly, the pollutants on the membrane surface can be effectively degraded under light radiation to restore its underwater superoleophobicity, showing the unique self-cleaning ability of the membrane. In view of its good self-cleaning ability and environmental stability, the membrane can be used for wastewater treatment and oil spill recovery and has a broad application prospect in water treatment in complex environments.

## 1. Introduction

With the improvement of industrialization, a large number of industrial activities and oil spill accidents have caused serious oil pollution. Large amounts of oily wastewater cause serious damage to our aquatic ecosystems and indirectly affect human health and well-being [1,2,3,4]. Traditional oil-water separation methods such as gravity separation [5], centrifugation [6], flotation [7], the electric field method [8], and so forth, have been used to solve the treatment of oil-containing wastewater. However, there are still some deficiencies, such as low separation efficiency, high energy consumption, and secondary pollution [9]. Therefore, it is urgent to develop an efficient treatment method to solve the problem of oil-water separation, especially for oily wastewater in complex environments.

Recently, membrane technology has made great progress in the field of oil-water separation [10,11,12,13,14]. Compared with the traditional oil-water separation methods, the membrane separation process has the following advantages: low energy consumption, a small footprint, low pollution, and efficient separation [15,16,17,18,19]. Oil-water separation membranes, such as super-hydrophobic/super-lipophilic membranes, can effectively separate oily wastewater, but the pores of lipophilic membranes are easily blocked by oil during the separation process, leading to a reduction in flux and service life. In contrast, super-hydrophilic/underwater super oleophobic membranes can adsorb water to form a hydrated layer and simultaneously repel oil, showing more advantages in oil-water separation, so the super-hydrophilic/underwater super-oleophobic membranes have attracted much attention and research from many researchers. For instance, Helali et al. [20] synthesized polyamide-imide microfiltration (PAI MF) membranes by non-solvent-induced phase separation techniques. This work has achieved effective results in the separation of oil-water emulsions and provided a new paradigm for the application of PAI MF membranes in oily wastewater treatment in a broad range of commercial processes. Fan et al. [21] prepared hydrogel-coated filter paper using glutaraldehyde as a crosslinking agent through a simple aldol condensation reaction, and the hydrogel-coated filter paper not only can separate oil-water mixtures in highly acidic, alkaline, and salty environments but also separate surfactant-stabilized emulsions. Wei et al. [22] prepared an underwater oleophobic PTFE membrane by UV-initiated reaction, which achieved high efficiency, high flux, and long-term stability in the separation process of oil-in-water emulsion. Cui et al. [23] prepared a crosslinking modified PVDF/GO membrane by a simple crosslinking process between acrylic acid and ethylene glycol dimethacrylate for oil-water emulsion separation. Kallem et al. [24] fabricated a composite membrane substrate by adding PDA@TiO_2_ to polyether sulfone through a non-solvent-induced phase separation process. The as-prepared membrane shows enhanced permeability and fouling resistance in the treatment of oily wastewater. Peng et al. [25] prepared a novel super-hydrophilic/underwater super-oleophobic polyacrylonitrile ultrafiltration membrane by a hydroxylamine-induced phase inversion process, exhibiting superior antifouling properties and oil-water separation properties for various oil-in-water emulsions. Various super-hydrophilic membranes have been successively reported for oil-water separation, showing excellent water permeability, selectivity, and good mechanical properties. Compared with organic-modified hydrophilic membranes, inorganic-modified hydrophilic membranes show more prominent advantages in terms of physico-chemical performances and mechanical stability. Such inorganic compounds (SiO_2_ [26,27], ZrO_2_ [28], ZnO [29], and so on.) have been successively applied in oil-water separation, and they have shown excellent performance and mechanical stability. However, the treatment of oily sewage in a complex environment is undoubtedly a great test for the conventional inorganic-modified membrane. On the other hand, in the long-term process of oil-water separation, a small amount of oil will inevitably attach to the surface of the membrane, resulting in the loss of the original special wettability of the membrane and affecting its separation performance. For membrane pollution issues, some inorganic materials with catalytic activity (such as TiO_2_ [30], WO_3_ [31], Bi_2_WO_6_@CuO [32], etc.) have been gradually applied to the oil-water separation membrane modification. Among them, TiO_2_ has been widely studied in modifying membranes because of its hydrophilicity, economic, non-toxic, and catalytic self-cleaning properties [33,34,35,36]. Zhang et al. [37], who prepared an electrospun stereocomplex polylactide membrane using a GA-modified TiO_2_ coating, which showed superhydrophilicity and underwater superoleophobicity in various harsh working conditions and exhibited efficient separation properties for a wide range of oil/water mixtures and oil-in-water emulsions. Nakamoto et al. [38] prepared a self-cleaning TiO_2_ coating-modified PAPS/PBE membrane, which showed excellent photocatalytic performance and provided a new idea for a self-cleaning membrane. Salehian et al. [39] synthesized photocatalytic TiO_2_@MIL-88A(Fe)/polyacrylonitrile mixed matrix membranes, which effectively improved the anti-pollution performance of the membrane in the process of oil-water separation. The above membranes show excellent super-hydrophilic/underwater super-oleophobic performance and good oil-water separation performance. However, the existing preparation methods for TiO_2_-modified membranes have the disadvantages of a complex operation process, low coating coverage, and high instrument requirements, which severely limit their wide application. In addition, the organic matrix is still greatly hindered in complex environments because of its unstable physicochemical properties, whereas most of the inorganic matrix will have no such obstacles. High silicoxy cloth is a kind of high-temperature-resistant inorganic fiber with a silicon dioxide (SiO_2_) content higher than 96% that has the characteristics of high strength and easy processing. It is often used as a high-temperature and ablation-resistant material and has excellent corrosion resistance. Therefore, using high-silica fiber cloth (recorded as SiO_2_ fiber membrane) as the substrate is not only suitable for the treatment of oily wastewater in a complex environment but also conducive to the construction of the hierarchical structure. As far as we know, there is no research on TiO_2_-modified SiO_2_ fiber membranes for oil-water separation in acid, alkali, and salt environments.

In this work, we prepared a self-cleaning TiO_2_/SiO_2_ fiber membrane (TSFM) by the hydrothermal method for oil-water separation in acid, alkali, and salt environments. The effect of TiO_2_ nanoparticle attachment on the surface wettability and separation properties of TSFM was studied. The TSFM displays excellent superhydrophilicity/underwater superoleophobicity and self-cleaning ability and shows excellent selective separation for various oil-water mixtures in different environments. In addition, contaminated TSFM after long-term oil-water separation can restore its wetting performance and excellent selective separation capacity when the membrane surface is exposed to light. In view of its good self-cleaning ability and environmental stability, the TSFM has broad application prospects in water treatment in complex environments.

## 2. Experimental Methods

### 2.1. Materials

SiO_2_ fiber membrane (SFM, thickness: about 260 μm) was provided by Jinhe New Materials Co., Ltd. (Ningbo, China), tetrabutyl titanate (TBOT), anhydrous ethanol, concentrated hydrochloric acid (HCl, 36–38%), petroleum ether, n-hexane, dichloroethane, liquid paraffin, sodium chloride, sodium hydroxide, and Sudan red were bought from the Shanghai Aladdin Biochemical Technology Co. Ltd. (Shanghai, China). Deionized (DI) water was used in this work.

### 2.2. Preparation of TSFM

The SiO_2_ fiber membranes (3 cm × 3 cm) were placed in an ethanol solution with ultrasonic treatment for 30 min to remove residual impurities on the surface, then washed three times with deionized water and dried at 50 °C.

**Figure 1:**
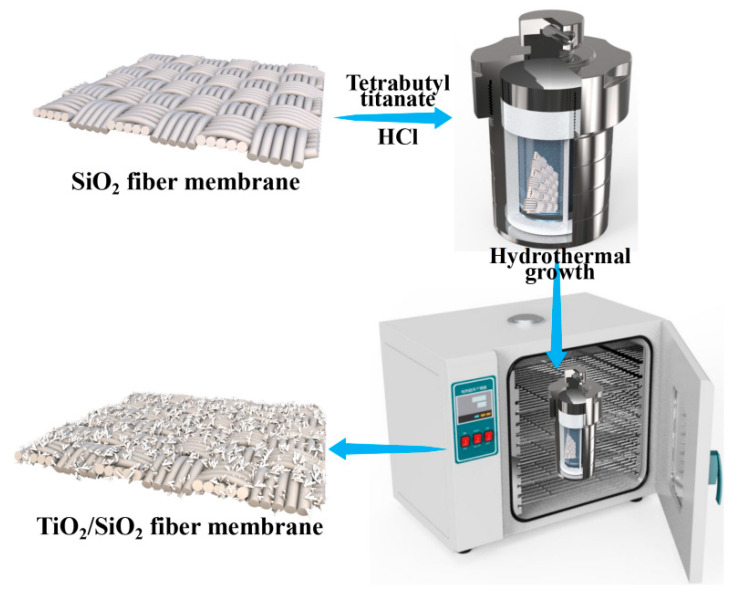
Schematic diagram of the preparation process for TSFM.

Based on pioneering research, for instance, Yuan et al. [40] studied the effect of hydrothermal temperature on the morphology of TiO_2_, and the results showed that TiO_2_ nanotubes were generated when the temperature was between 100 and 180 °C. Beyond this range, the number of nanotubes decreased. Seo et al. [41] found that the length and quantity of TiO_2_ increased as the temperature increased. Moreover, the prepared nanotubes exhibit superior properties at 150 °C. Here, 150 °C was chosen as the hydrothermal reaction temperature. The TSFM was prepared by a hydrothermal method, as shown in Figure 1. Tetrattyl titanate of 1 mL was added to a HCl solution (6 mol/L) of 30 mL with magnetic stirring for 10 min, and the mixed solution was transferred to a stainless-steel autoclave lined with polytetrafluoroethylene to soak a SiO_2_ fiber membrane, sealed, and maintained for different reaction times (1 h, 3 h, 5 h, and 9 h) at 150 °C. After the hydrothermal reaction was finished and cooled to room temperature, the TSFM-*x* (*x* represents hydrothermal time) was removed from the stainless-steel autoclave, washed with deionized water, and dried naturally.

### 2.3. Oil-Water Separation Tests

The water pre-infiltrated TSFM was fixed between two glass devices. The mixture of oil and water (dichloroethane, n-hexane, petroleum ether, and liquid paraffin) (V:V = 1:1) was slowly poured into the glass tube. The permeated liquid was collected in a beaker. During separation, water rapidly passed through the membrane, and the oil was blocked by the membrane. Then, the separation efficiency (*E*) of the oil-water mixture is calculated according to the following formula:

(1) $${E=m}_{0}/m_{1}\times 100\%$$

where *m*_0_ (g) and *m*_1_ (g) are the weights of water before and after separation, respectively.

Permeation flux (*F*) is calculated by the following formula:

(2) F=V/(A×Δt)

where *V* stands for the liquid filtration volume (L), *A* represents the effective filtration membrane area (m^2^), and Δ*t* is the filtration time (h).

### 2.4. Mechanical Stability Tests

The effects of the fluid shear force and impact force on the membrane properties were tested experimentally. Specifically, as follows: (1) A TSFM was putted in water and stirred at 1000 rpm for 12 h; (2) A TSFM was fixed on a glass sheet, and water was impacted on the TSFM surface at a height of 50 cm for 2 h. The surface wettability and separation properties were measured after treatment.

### 2.5. Environmental Stability Tests

The membrane was immersed in sodium chloride (1 mol/L), sodium hydroxide (1 mol/L), and hydrochloric acid (1 mol/L) for 12 h, and then the environmental stability of the membrane was evaluated by testing the underwater oil contact angle and oil-water separation performance.

### 2.6. Anti-Fouling and Self-Cleaning Performance Tests

The oil-water separation was repeatedly conducted for 20 times, and the underwater oil contact angle of the used membrane was determined. The TSFM was immersed in appropriate water and exposed to a xenon lamp for 1 h to study the changes in underwater oil contact angle before and after irradiation.

### 2.7. Characterizations

The surface morphology and EDS mapping of the membranes were studied by scanning electron microscopy (SEM, 400FEG, FEI, Hillsboro, OR, USA). The chemical composition of the membrane was analyzed by X-ray photoelectron spectroscopy (XPS, Thermo ESCALAB 250XI, USA). The phase structure of samples was analyzed by X-ray diffraction (XRD, XRD-7000, Shimadzu, Kyoto, Japan). The water contact angle and underwater oil contact angle (UOCA) were tested by a contact angle measurement system (OSA60, LAUDA Scientific, Baden-Württemberg, Germany).

## 3. Results and Discussion

### 3.1. Characterization of the Membranes

**Figure 2:**
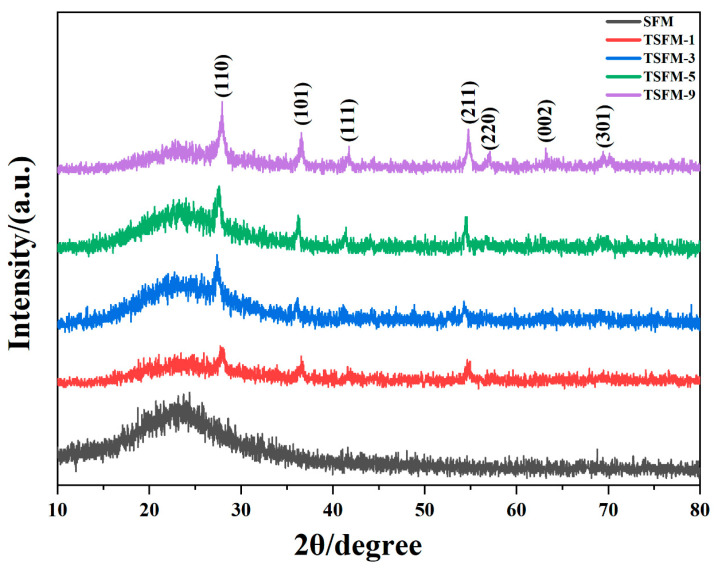
The XRD diffraction patterns of the SiO_2_ fiber membranes, TSFM-*1*, TSFM-*3*, TSFM-*5*, and TSFM-*9*.

The phase structure of the samples was analyzed by XRD. Figure 2 shows the XRD diffraction patterns of the SiO_2_ fiber membranes, TSFM-*1*, TSFM-*3*, TSFM-*5*, and TSFM-*9*. The diffraction pattern of the SiO_2_ fiber membrane appears as a broad diffraction peak at 2θ = 22°, corresponding to the crystal face of silica (222). The distinct diffraction peaks appear at 2θ = 27.1°, 35.8°, 54.0°, 56.3° in the diffraction pattern of all modified membranes, corresponding to the (110), (101), (211), and (220) crystal planes of the rutile phase. [42,43] As the hydrothermal time increases, the diffraction peak intensity of the TSFM gradually increases. The diffraction peak of the (110) crystal plane is significantly enhanced, indicating the growth of the nanorods on the (110) speed is greater than in other directions. The XRD analysis showed that TiO_2_ nanoparticles in the rutile phase were successfully grown on the surface of TSFM.

**Figure 3:**
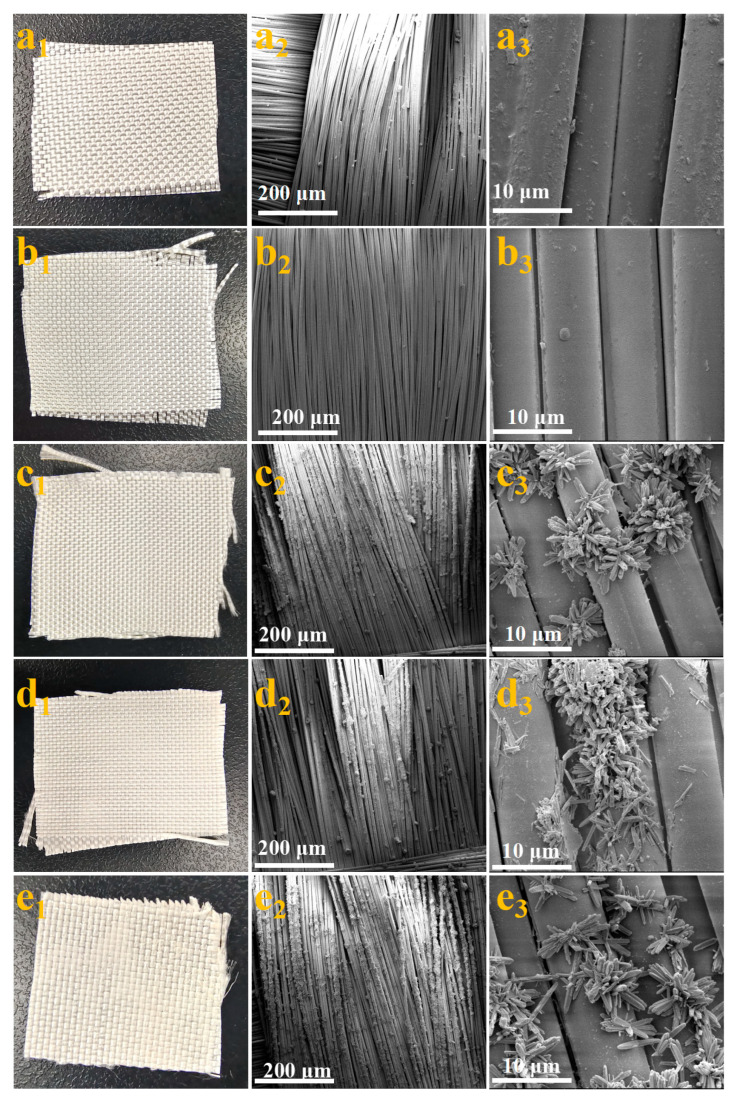
Photographs and SEM images of (**a_1_**–**a_3_**) SiO_2_ fiber membrane, (**b_1_**–**b_3_**) TSFM-*1*, (**c_1_**–**c_3_**) TSFM-*3*, (**d_1_**–**d_3_**) TSFM-*5*, and (**e_1_**–**e_3_**) TSFM-*9*.

Photographs of the SiO_2_ fiber membranes TSFM-*1*, TSFM-*3*, TSFM-*5*, and TSFM-*9* are shown in Figure 3(a_1_–e_1_). It can be observed that the white matter growing on the surface of the fiber membrane gradually increased with the increase in hydrothermal time. The SEM images of the SiO_2_ fiber membranes TSFM-*1*, TSFM-*3*, TSFM-*5,* and TSFM-*9* are shown in Figure 3(a_2,_a_3_–e_2,_e_3_). As can be observed, the SiO_2_ fiber membrane has a porous network structure composed of fibers, and the fibers show a relatively smooth surface with an average diameter of 6–7 μm. As shown in Figure 3(b_2,_b_3_), a layer of nano-sized TiO_2_ seeds was obviously grown on the surface of TSFM-*1*. With the extended hydrothermal time, a flower-like hierarchical structure assembled by TiO_2_ nanorods was successfully constructed on the fiber’s surface, and the diameter and length of TiO_2_ nanorods increased slightly with the increase in hydrothermal time. The observed results indicated that a layer of a flower-like hierarchical structure was successfully constructed on the surface of the SiO_2_ fiber membrane by in situ hydrothermal growth. This flower-like hierarchical structure is very beneficial for the construction of super-hydrophilic surfaces.

**Figure 4:**
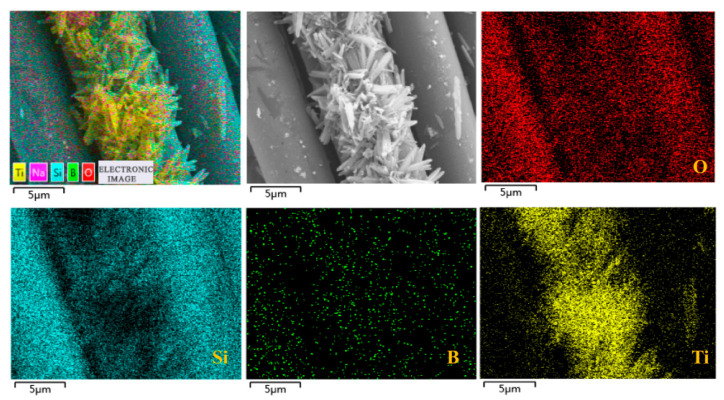
Elements mapping images of the TSFM.

Energy dispersive X-ray spectroscopy (EDS) was used to study the surface elemental composition and distribution of TSFM. The results are shown in Figure 4. We can see the existence of the elements Si, O, and B in images, which are consistent with the composition of the information (Na_2_O-B_2_O_3_-SiO_2_) provided by the manufacturer. These basic elements are evenly distributed on the surface of fibers. In addition to the elements from the SiO_2_ fiber membrane itself, the Ti element was also detected on the surface of the fibers. The distribution of the Ti element matches the observed TiO_2_, which preliminarily proved that the TiO_2_ nanorod was successfully grown on the fiber surface.

**Figure 5:**
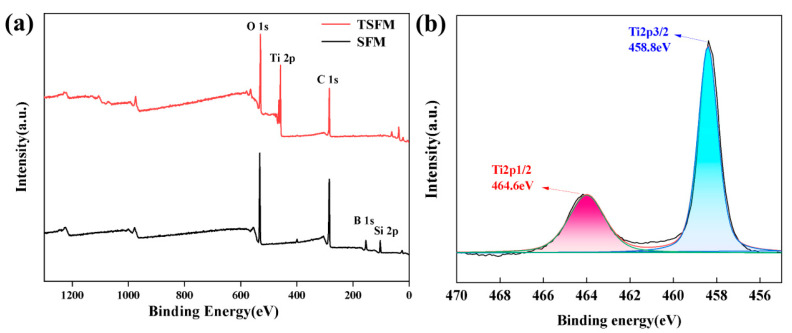
(**a**) The survey XPS spectra of SiO_2_ fiber membrane and TSFM; (**b**) The core-level XPS spectra of Ti 2p.

To further investigate the chemical composition of the membrane surface, the surface element bonding states were analyzed by XPS. The XPS spectra of the SiO_2_ fiber membrane and TSFM are shown in Figure 5. As seen, C 1s, O 1s, Si 2p, and B 1s signals were observed in the survey spectrum of the SiO_2_ fiber membrane (Figure 5a), and the atomic percentages are 62.20%, 27.22%, 8.58%, and 2.0% (Table 1), respectively. In addition to the C, O, Si, and B elements, new Ti 2p peaks also appear in the survey spectrum of the TSFM membrane, and the atomic percentages are 42.28%, 35.82%, 4.80%, 1.55%, and 15.55% (Table 1), respectively. The core-level spectrum of Ti 2p (Figure 5b) can be fitted by two peaks at 458.8 eV and 464.6 eV, which are attributed to Ti 2p_3/2_ and Ti 2p_1/2_ [44], respectively. The results of the XPS analysis confirmed the successful growth of TiO_2_ on the surface of the fiber membrane, which was consistent with the results of the XRD and EDS analyses.

### 3.2. Surface Wettability of Membranes

**Figure 6:**
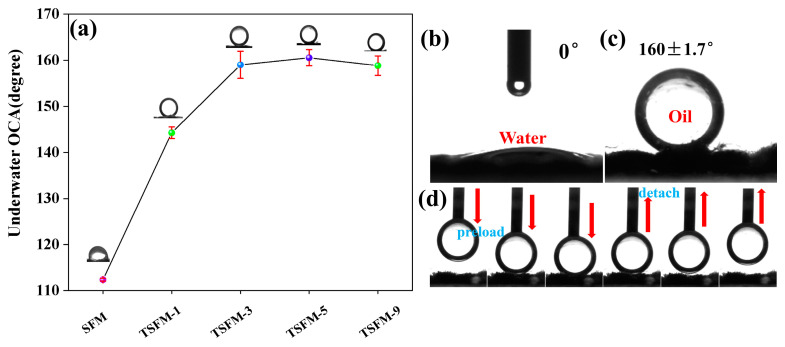
(**a**) UOCA for the different membranes; (**b**) WCA of TSFM; (**c**) UOCA of TSFM; (**d**) Dynamic underwater oil adhesion process of TSFM.

The wettability of membrane is a key factor affecting the oil-water separation process, which is directly related to the separation capability and antifouling ability of membrane. The surface wettability of the membrane was assessed by measuring the underwater oil contact angle (UOCA, using dichloroethane as the testing oil) and water contact angle (WCA). As shown in Figure 6a, the UOCA value of the original SiO_2_ fiber membrane was only 112.4°, indicating that the original fiber membrane had poor underwater oleophobic performance. With the increase in hydrothermal time, the UOCA of TSFM gradually increased from 112° to 160°, but when the hydrothermal time reached 9 h, the UOCA was slightly reduced. From Figure 6b, a water droplet fully spreads and penetrates onto the surface of the TSFM with a WCA of about 0°. In addition, a dichloroethane droplet on the TSFM surface underwater is spherical with a UOAC of 160 ± 1.7° (Figure 6c), indicating that the TSFM is super hydrophilic/underwater super oleophobic. The dynamic oil adhesion process of underwater oil droplets on the membrane surface was measured to evaluate the adhesion force between the oil droplet and the membrane surface (Figure 6d). An oil droplet was gradually pressed on the surface of TSFM, and then the oil drop was removed from the membrane surface. The oil droplet did not deform due to the small adhesion force at the moment when it left the membrane surface. The results indicated that the TSFM surface has low oil adhesion, which is beneficial for reducing the adhesion of oil droplets on the membrane surface during the oil-water separation process.

### 3.3. Oil-Water Separation Performance

**Figure 7:**
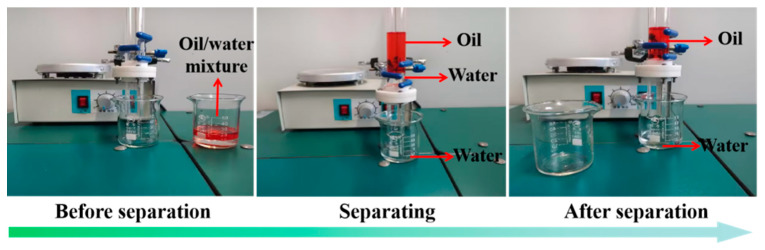
Photographs the separation process of petroleum ether-water mixture.

The pre-infiltrated TSFM with water was fixed between the two glass devices. Take the separation of a petroleum ether-water mixture (petroleum ether was stained with Sudan red) as an example. The mixture was slowly poured into the glass tube, and the penetrating liquid was collected with a beaker. During the separation process, water quickly passed through the membrane, while oil was blocked by the membrane. As shown in Figure 7, there is no visible petroleum ether stained with Sudan red in the filtrate after separation. These results showed that TSFM can effectively separate the oil-water mixture.

**Figure 8:**
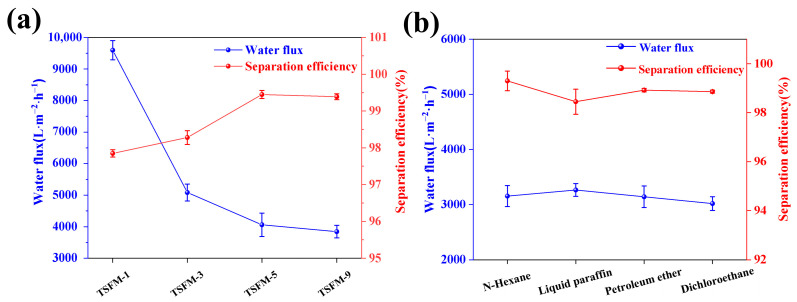
(**a**) The oil-water separation efficiency and permeation flux of TSFM-*1*, TSFM-*3*, TSFM-*5,* and TSFM-*9*, (**b**) The separation efficiency and permeation flux for different oil-water mixtures.

Figure 8a shows the separation efficiency and permeation flux of TSFM-*1*, TSFM-*3*, TSFM-*5,* and TSFM-*9* for the petroleum ether-water mixture (due to the poor underwater hydrophobicity, the original SiO_2_ fiber membrane cannot separate the oil-water mixture, so no data were provided). With the increase in hydrothermal time, the separation efficiency of membranes increased from 97.85% to 99.40%, while the permeation flux decreased from 9596.85 L·m^−2^·h^−1^ to 3843.38 L·m^−2^·h^−1^. This may be related to the growth of TiO_2_ on the surface of the fiber membrane because the growth of TiO_2_ reduced the pore size of the fiber membrane and impeded the penetration of water. Considering the practical application performance and cost, the optimized TSFM-*5* (*abbreviated as TSFM unless otherwise stated*) was taken as a representative sample for further testing.

The separation performance of TSFM for different oil-water mixtures was tested experimentally. Figure 8b shows the separation efficiency and permeation flux of TSFM for various oil-water mixtures. The results indicated that the separation efficiencies of TSFM were higher than 98.45% for different oil-water mixtures. The permeation fluxes for n-hexane, liquid paraffin, petroleum ether, and dichloroethane-water mixtures were 3135.06 L·m^−2^·h^−1^, 3263.45 L·m^−2^·h^−1^, 3140.42 L·m^−2^·h^−1^ and 3016.38 L·m^−2^·h^−1^, respectively. The differences in separation efficiency and flux may be caused by the different properties of various oils, such as density, viscosity, and so on (Table 2).

### 3.4. Stability and Anti-Fouling of Membranes

**Figure 9:**
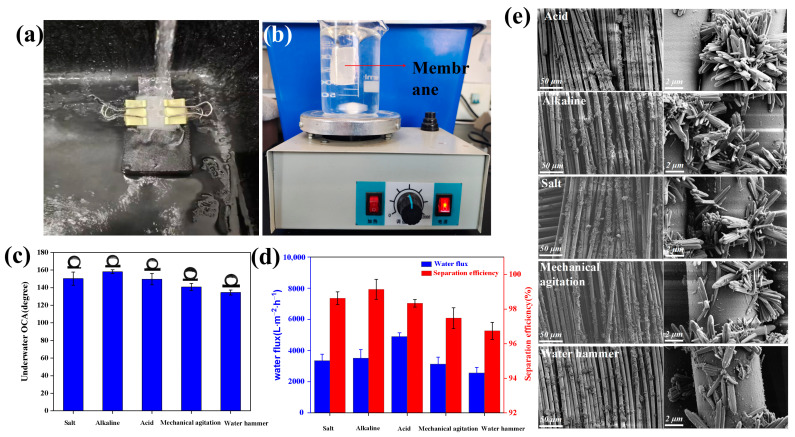
Mechanical stability testing: (**a**) water impact; (**b**) mechanical agitation; (**c**) UOCA of TSFM after different treatments; (**d**) separation performance of different treatments; (**e**) SEM images of TSFM after the stability test.

The mechanical stability of the surface microstructure is an important factor in determining the service life of the membrane. Many external influences caused by water flow, such as shear forces and impact forces, can destroy the surface microstructure and membrane separation function. To assess mechanical stability, the effects of mechanical agitation and water impact tests on TSFM were tested. (Figure 9a,b), under the action of external force, the UOCA of TSFM decreased to a certain extent [45], but it still has a good separation performance of 96.7% for the oil-water mixture (Figure 9b,d). The above results indicated the good mechanical stability of the membrane.

In practical application, environmental stability plays a crucial role in the separation performance and service life of membranes, which determine whether membranes can be continuously used in complex environments. Generally, inorganic-modified separation membranes such as oxides and metals are unstable in aqueous solutions containing strong acids or bases [46,47]. While the organic membranes are easily destroyed in organic solvents (such as 1,2-dichloromethane, chloroform, diesel, etc.) [48,49], which seriously affected their practical application. To assess the environmental stability, the TSFM was put into sodium chloride (1 mol/L), sodium hydroxide (1 mol/L), and hydrochloric acid (1 mol/L) solutions for 12 h [50], and then the environmental stability of the membrane was evaluated by testing the UOCA and oil-water separation efficiency. As shown in Figure 9c, the UOCA of the membrane was maintained above 150°, indicating the membrane surface can still maintain underwater superoleophobicity. At the same time, the separation efficiency of the petroleum ether-water mixture was still higher than 98%, and the separation flux remained above 3357 L·m^−2^·h^−1^ (Figure 9d). The surface morphology of samples before and after the stability test (Figure 9e) was compared. The results showed that the morphology of TiO_2_ particles on the membrane surface was not significantly damaged after the chemical stability test, indicating that it still had excellent chemical stability. However, the TiO_2_ particles on the membrane surface were subjected to strong external forces in the mechanical stability test, resulting in a slight decrease in TiO_2_ adhesion on the fiber surface but still maintaining a certain level of performance. This explains why the separation efficiency of the membrane slightly decreases after mechanical stability testing. The above results showed that the membrane has good stability in complex environments.

**Figure 10:**
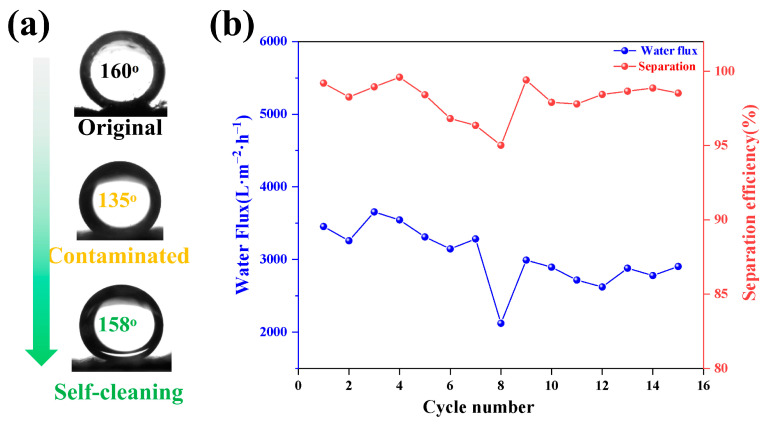
The UOCA (**a**) and separation performance (**b**) of cyclic separation test process.

Another problem faced by membrane materials in the application process is membrane fouling. After continuous separation of complex oily wastewater, the membrane may be contaminated by some oil or organic molecules, leading to the loss of the original surface wettability and separation ability. In order to study the anti-fouling performance, the repeated separation performance of the TSFM for the petroleum ether-water mixture was studied experimentally. The changes in UOCA and separation performance of the membrane are shown in Figure 10. Before separation, the original UOCA was 160°. As the number of times the separation process was repeated increased, the separation efficiency decreased to 95.02% at the 8-cycle experiments, which indicated the TSFM was polluted to some extent, and the UOCA was reduced to 135°. The contaminated TSFM was irradiated under a xenon lamp for 1 h, the UOCA was restored to 158°, and the separation efficiency was significantly improved to 99%. The results showed that TSFM has excellent anti-fouling and self-cleaning properties.

### 3.5. Separation Mechanisms

**Figure 11:**
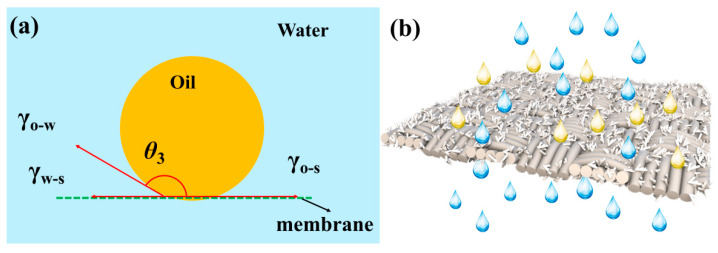
(**a**) Solid/water/oil interface model, (**b**) schematic diagram of oil-water separation.

Hierarchical structure and surface energy are two key factors for constructing super-wettability materials. According to the Wenzel equation (*cosθ_w_* = *rcosθ*) [51], the hierarchical structure can significantly enhance the wettability of the surface. After introducing the hierarchical structure, the capillary effect enhances the surface wettability. As shown in Figure 11a, a new solid/water/oil interface was formed when the oil droplets came into contact with the membrane. The wetting state of underwater oil droplets on TSFM can be explained by Young’s equation [52]:

(3) $${\cos\theta }_{3}=\frac{\gamma_{o-g}{\cos\theta }_{1}\;-\;\gamma_{w-g}{\cos\theta }_{2}}{\gamma_{o-w}}$$

where *θ*_1_ represents the contact angle of oil in the air, *θ*_2_ represents the contact angle of water in the air, *θ*_3_ represents the contact angle of oil in water, *γ* represents the surface tension, and subscripts *o*, *w*, and *g* represent the oil, water, and gas phases, respectively. It can be used to calculate the underwater superoleophobicity of TSFM. The measurement results of CA indicated the *θ*_1_ = 0°, *θ*_2_ = 0°, thus *cosθ*_1_ = 1, *cosθ*_2_ = 1. From the data in Table 2, ${\cos\theta }_{3}=\frac{\gamma_{o-g}\;-\;\gamma_{w-g}}{\gamma_{o-w}}$ < 0 (*γ_o−w_* > 0), that is ${\cos\theta }_{3}$ < 0. Therefore, it is calculated that *θ*_3_ value must be greater than 90°, which means the TSFM is oil-repellent underwater. As shown in Figure 11b, water permeates the TSFM, and oil is rejected by the membrane, successfully achieving the separation of the oil-water mixture.

## 4. Conclusions

The TSFM was successfully prepared by a one-step hydrothermal method for oil-water separation in acid, alkali, and salt environments. The membrane showed superhydrophilicity/underwater superoleophobicity with a WCA of 0° and a UOCA of 160°. The optimal TSFM exhibited excellent separation performance, with a separation efficiency of above 98%. Furthermore, the optimal TSFM showed excellent mechanical resistance to fluid shear/impact forces and environmental resistance to alkali, acid, and salt. Moreover, the TSFM exhibited anti-fouling self-cleaning ability, ensuring long-term separation performance. In a word, a simple one-step method for fabricating super-hydrophilic/underwater super-oleophobic membranes with good separation performance and excellent anti-fouling self-cleaning ability was reported in this work.

## Figures and Tables

**Table 1:** Surface chemical composition (at.%) of membranes examined by XPS.

Elements (at.%)	C	O	B	Si	Ti
SiO_2_ fiber membrane	62.20	27.22	2.0	8.58	/
TSFM	42.28	35.82	4.80	1.55	15.55

**Table 2:** Summary of the properties of the oils and water.

Liquids	The Viscosity of Oils (mPa s)	Density (g cm^−3^)	Surface Tension (mN m^−1^)
Liquid paraffin	14.2–17.2	0.86–0.91	33.1
n-hexane	0.33	0.66	18.4
Petroleum ether	0.3	0.66	18.8
dichloroethane	0.84	1.245	32.2
Water	0.89	1	72.8

## Data Availability

Not applicable.
